# Dr. William S. McDowell

**Published:** 1898-01

**Authors:** 


### Obituary.

### DR. WILLIAM S. McDOWELL.

Dr. William S. McDowell, a well-known dentist, died January 2nd, 1898, at his home, 1005 West Lafayette avenue. He had been in failing health for about five years. About that time he fell on the ice and broke his hip, and later retired from active service. The cause of death was pneumonia, together with a complication of diseases.

Dr. McDowell was born in Philadelphia and was seventy-six years old. He commenced practice in Philadelphia and New York City in 1854, and soon after came to Baltimore to practice. His contemporaries were Dr. Chapin Harris and Dr. C. O. Cone, the latter of whom he succeeded in practice in 1858. He had patients from Massachusetts to Cuba. At the beginning of the war he was interested with other Union men in retaining Maryland in the Union, and was one of the organizers of the Union League, the first organization of its kind in the country. He was elected its first president.

Dr. McDowell was an able and conscientious dentist, a prominent member of the Baptist Church, and a respected citizen in the community where, for so long a time he practised the profession of dentistry.

The character of his operations were always an evidence of his professional skill and ability.

